# Transcranial magneto-acoustic stimulation improves spatial memory and modulates hippocampal neural oscillations in a mouse model of Alzheimer's disease

**DOI:** 10.3389/fnins.2024.1313639

**Published:** 2024-02-07

**Authors:** Shuai Zhang, Zhongsheng Guo, Yihao Xu, Jinrui Mi, Jun Liu, Zichun Li, Xiaofeng Xie, Guizhi Xu

**Affiliations:** ^1^State Key Laboratory of Reliability and Intelligence of Electrical Equipment, Hebei University of Technology, Tianjin, China; ^2^Hebei key Laboratory of Bioelectromagnetism and Neural Engineering, Hebei University of Technology, Tianjin, China; ^3^Tianjin Key Laboratory of Bioelectromagnetic Technology and Intelligent Health, Tianjin, China

**Keywords:** transcranial magneto-acoustic stimulation, neuromodulation, Alzheimer's disease, neural oscillations, local field potentials

## Abstract

**Introduction:**

In our study, we applied transcranial magneto-acoustic stimulation (TMAS), a technique based on focused ultrasound stimulation within a static magnetic field, in the APP/PS1 mouse model of Alzheimer's disease (AD) to explore the feasibility of TMAS on improving AD related spatial memory deficits and abnormal neural oscillations.

**Methods:**

The mice treated with TMAS once daily for 21 days. We recorded local field potential signals in the hippocampal CA1 region of the mice after TMAS treatment with *in-vivo* electrophysiology and evaluated the neural rehabilitative effect of TMAS with sharp-wave ripple (SWR), gamma oscillations during SWRs, and phase-amplitude coupling (PAC). The spatial memory function of the mice was examined by the Morris water maze (MWM) task.

**Results:**

We found that TMAS improved the performance of MWM related spatial cognitive functions compared with AD group. Furthermore, our results implied that TMAS alleviated abnormalities in hippocampal SWRs, increased slow gamma power during SWRs, and promoted theta-slow gamma phase-amplitude coupling. These findings suggest that TMAS could have a positive influence on spatial memory through the modulation of neural oscillations.

**Discussion:**

This work emphasizes the potential of TMAS to serve as a non-invasive method for Alzheimer's disease rehabilitation and promote the application of TMAS for the treatment of more neurological and brain aging diseases in the future.

## 1 Introduction

As global life expectancy continues to increase, the world's aging population is growing. Concurrently, the prevalence of neurodegenerative disorders, including dementia, is also increasing (Livingston et al., 2020). Among these conditions, Alzheimer's disease (AD) emerges as the most common form of dementia, primarily affecting the elderly (Dolgin, 2016). AD is a neurodegenerative disorder that leads to cognitive impairments and memory dysfunction (Wu et al., 2022). The deposition of amyloid beta and the formation of neurofibrillary tangles are the pathological processes associated with AD (Yokoyama et al., 2022). These pathological features have the potential to disrupt synaptic and neuronal activity, causing network abnormalities in various brain regions (Casula et al., 2022; Luo et al., 2023; Pless et al., 2023). In the brains of AD patients, various neurophysiological features have been detected, including hyperexcitability in the precuneus cortex (Casula et al., 2023) and impairment of cerebellar-cortical plasticity mechanisms (Di Lorenzo et al., 2020). These abnormal neural activities may lead to neuronal network dysfunction in AD, thereby contributing to cognitive impairment. The hippocampus, a critical brain region for memory encoding, storage, and retrieval, is among the earliest regions affected by AD pathology (Gillespie et al., 2016; Caccavano et al., 2020). Researchers have detected anomalies in neural oscillations that are linked to with cognitive processes involved in memory by using electroencephalogram or local field potential (LFP) recordings in the hippocampal region of both AD patients and animal models (Roux and Uhlhaas, 2014; Miller et al., 2018; Jafari and Kolb, 2020; Zhou et al., 2022). Further exploration of their role within the context of AD pathology has revealed potential opportunities for interventions in the treatment of AD (Chan et al., 2021; Traikapi and Konstantinou, 2021).

The hippocampus contains a significant population of interneurons that play a crucial role in driving neuronal synchronization (da Cruz et al., 2020; He et al., 2021). Gamma oscillations within the hippocampus have been associated with memory and cognition in both animals and humans, and it is possible that functional distinctions exist across various frequency ranges (Mably and Colgin, 2018). Specifically, slow gamma oscillations (25 Hz−50 Hz) are thought to enhance memory retrieval processes within the hippocampus (Zheng et al., 2016), with increased slow gamma activity being observed during tasks involving higher memory demands (Rangel et al., 2016). Hippocampal sharp-wave ripple (SWR) plays an important role in supporting memory consolidation and replay (Buzsaki, 2015; Katsuki et al., 2022). Disruption of SWR can impair memory performance (Aleman-Zapata et al., 2022), while prolonging the duration of SWR through optogenetic stimulation improves memory performance in rats during maze tasks (Fernández-Ruiz et al., 2019). Research has revealed defects in hippocampal gamma oscillations and SWR in AD (Hollnagel et al., 2016; Klein et al., 2016; Witton et al., 2016; Benthem et al., 2020).

Neural stimulation is a method of neuroregulation that involve delivering stimulations, such as electrical, magnetic, optical, and ultrasound, to selected brain areas in order to modulate local and network-wide neuronal activity (Yuan et al., 2020). Transcranial magneto-acoustic stimulation (TMAS) is an innovative form of a non-invasive tool that allows for the stimulation of specific brain regions within a static magnetic field using low-intensity focused ultrasound (Yuan and Chen, 2016; Wang et al., 2019). In 2003, Norton proposed the idea of using ultrasounds for stimulation in a static magnetic field (Norton, 2003). The motion of ionic particles induced by ultrasounds inside brain tissue will form Lorentz force under a static magnetic field, and TMAS allows the combined action of a magneto-acoustic electric field and an ultrasound wave (Wang et al., 2016; Yuan et al., 2016). Notably, even in deep brain regions, TMAS can provide a high spatial resolution stimulating electric field at the target site due to the utilization of focused ultrasound (Liu et al., 2019; Yu et al., 2021). TMAS possesses distinct advantages that address the demands of depth and improved localization, holding significant research value and promising potential applications in the development of intervention techniques for brain functional disorders (Chu et al., 2023).

Research indicates that TMAS, as a non-invasive neurostimulation approach, can modulate neuronal activity to enhance brain function (Liu et al., 2019; Wang et al., 2019; Zhang et al., 2022). In this study, we applied TMAS to treat AD model mice model and examined its impact on neuronal activity in the hippocampus. To assess the impact of TMAS on spatial memory in AD mice, we conducted Morris water maze (MWM) tests. Additionally, we recorded *in vivo* electrophysiological signals of the mouse hippocampus to explore potential underlying mechanisms. Our findings demonstrate that TMAS can improve spatial memory in AD mice and regulate hippocampal oscillations, providing a promising intervention approach for AD treatment.

## 2 Materials and methods

### 2.1 Animals

The animal model for Alzheimer's Disease (AD) in this study comprised 14 male APP/PS1 mice, aged 4 months, along with 7 age-matched male C57/BL6 mice (all purchased from Beijing HFK-Bio-Technology Co. Ltd., China). The animals were housed in the animal laboratory of Hebei University of Technology throughout the experimental period, with each mouse kept in a standard cage. The light cycle was maintained at 12 h (lights on at 7 am, off at 7 pm), and the housing environment was maintained at a temperature of 24°C and 50% humidity. Food and water were provided *ad libitum*. The 14 AD model mice were randomly divided into two groups of 7 mice each: the no-stimulation group and the TMAS group. Additionally, 7 C57 mice were included in the WT group. All experimental procedures were approved by Biomedical Ethics Committee of the Hebei University of Technology.

### 2.2 Operation

After sedating the mice with 2% isoflurane in the induction box, they were fixed onto a stereotactic frame (51670, Stoelting, USA). The mice's head was immobilized using ear bars and positioned horizontally, while 1%-2% isoflurane was administered via an anesthesia mask to maintain anesthetic state. Fur was removed from the scalp, the skin was cleaned with physiological 0.9% sodium chloride solution and sterilized with 70% ethanol. The scalp was incised along the midline of the skull to expose the bone, and the tissue was cleaned with 2% hydrogen peroxide. According to the stereotaxic map, a 1 mm square window was drilled through the skull for placing the recording electrode in the hippocampal CA1 region (AP: −1.82 mm, ML: 1.20 mm, DV: −1.30 mm). The electrode was then secured using dental cement in layers. After the surgery, mice were given a recovery period of at least 1 week.

### 2.3 TMAS treatment of AD model mice

**Figure 1:**
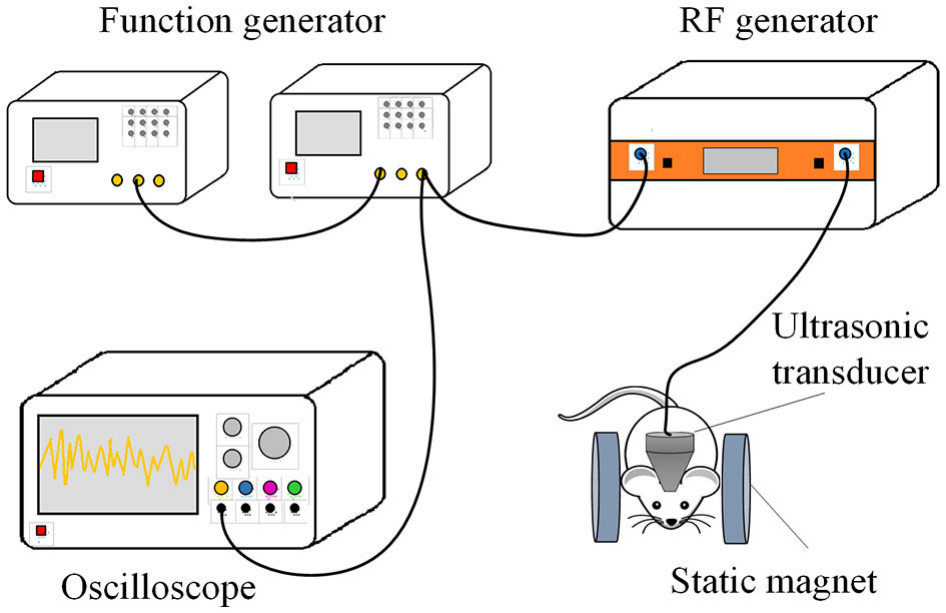
Diagram of transcranial magneto-acoustic stimulation system.

After surgical recovery, the TMAS group received 21 days of stimulation. The TMAS system, as depicted in Figure 1, consists of 2 function generators (33500B Series, KEYSIGHT, USA), a radiofrequency power amplifier (Model 150A100C, AR, USA), an ultrasonic transducer (P20FG, Shantou Electronics, China), an oscilloscope (TDS3014, Tektronix, USA), and 2 cylindrical neodymium iron boron permanent magnets. Two cylindrical neodymium iron boron permanent magnets with a diameter of 40 mm and thickness of 10 mm were employed to provide a horizontal static magnetic field of 0.3 T. The pulsed signals generated by two function generators were fed to the radiofrequency power amplifier and then sent to drive the ultrasonic transducer. The ultrasonic fundamental frequency was 0.5 MHz, the pulse repetition frequency was 1 kHz, the tone-burst duration was 0.5 ms, the sonication duration was 400 ms, the ultrasonic pressure was 0.3 MPa, the spatial-peak pulse-average intensity was 2.839 W/cm^2^, and the stimulation duration was 2 min. During stimulation, the mouse was anesthetized by 1% isoflurane, and TMAS was applied by connecting an ultrasound transducer through a conical collimator filled with bubble-free ultrasound coupling gel onto the mouse skull, targeting the hippocampal region. Mice of AD-TMAS group received TMAS once daily for a consecutive period of 21 days. Mice of AD groups received sham stimulation by keeping the turned-off ultrasound probe on the mouse head located within the same static magnetic field for the same amount of time as AD-TMAS groups.

### 2.4 Morris water maze test

The mice underwent 8 days of behavioral testing in the MWM. The round tank was divided into four equal quadrants by creating two invisible perpendicular lines. Each mouse was trained 4 times a day with an interval of at least 10 min between each session, during both the visible platform training and the hidden platform training. In the visible platform training, the platform was positioned at the center of one quadrant, elevated 0.5 cm to 1 cm above the water's surface. Mice were placed into water from the opposite quadrant, and their time to reach the platform and swimming speed were recorded using the Morris Water Maze video analysis system (SA201, SANS, China) to assess visual acuity and motor ability. If the mice did not find the platform after 60 s, they were guided to the platform and allowed to stay for 30 s−60 s. During the hidden platform training, the platform was kept in a constant position at the center of one quadrant and submerged 1 cm below the water's surface. The escape latency of mice was recorded, and if a mouse did not reach the platform within 60 s, it was guided to the platform, and the latency was recorded as 60 s. The probe trial was conducted 24 h after the completion of the hidden platform training. In this trial, the platform was removed, and each mouse underwent a 60-s test, during which the number of crossings through the target quadrant and the time spent in the target quadrant were recorded.

### 2.5 Electrophysiological recording

The electrophysiological signals of the mice were recorded for the subsequent 5 days after the completion of the entire stimulation period. Hippocampal electrophysiological activity was recorded using a 126-channel Plexon neural data acquisition system (Omniplex, Plexon Inc) while mice were in their home cages. Neurophysiological signals were collected at a frequency of 40 kHz through a headstage cable connected to the DigiAmp digital amplifier. LFP signals were stored on a PC and down-sampled to 1 kHz for offline analysis.

### 2.6 Data analysis

To detect SWR events, the local field potential (LFP) was bandpass filtered between 150 Hz−250 Hz, and the Hilbert transform was applied to obtain the SWR envelope amplitude. SWRs were identified as times when the envelope of the ripple-filtered trace exceeded 5 SDs of the signal for at least 15 ms (Cheng and Frank, 2008). The entire SWR was defined as the periods, containing times immediately before and after that prolonged threshold crossing event during which the envelope exceeded the baseline value. Analysis of SWRs was confined to periods of extended immobility. Spectrogram analysis was conducted using the multitaper method (Chronux toolbox) (Bokil et al., 2010). Spectrograms of SWRs were computed for a window extending 400 ms before and after the onset of each SWR. For evaluating cross-frequency coupling strength, the mean and standard deviation of the computed spectrograms were used to calculate z-score power for each frequency band. The quantification of slow gamma power during SWRs was calculated as the averaged the z-score power over a 30 Hz−50 Hz frequency band at 1 ms−100 ms after the initiation of SWR.

For the assessment of cross-frequency coupling strength, phase-amplitude coupling (PAC) was computed using the instantaneous phase and amplitude of the filtered LFP signal (Belluscio et al., 2012). Phase and amplitude in specific frequency bands were obtained through Hilbert transforms. The modulation index (MI) was calculated to quantify PAC between the theta band (4 Hz−12 Hz) and the slow gamma band (30 Hz−50 Hz).

### 2.7 Statistics

The results are presented as mean ± standard error of the mean (SEM). Data analysis was performed using the statistical software SPSS (IBM SPSS Statistics, IBM Corp., Armonk, NY, USA), and graph generation was carried out using GraphPad Prism software (GraphPad Software Inc., La Jolla, CA, USA). Levene's test was used to assess homogeneity of variances. For normally distributed data, one-way ANOVA was employed for comparison among multiple groups, followed by *post-hoc* evaluation using the Scheffe multiple range test. For non-normally distributed data, the Kruskal–Wallis test was used to assess differences in medians, and the Bonferroni method was applied for correcting multiple comparisons. *P* < 0.05 were considered statistically significant. ^*^P < 0.05; ^**^P < 0.01.

## 3 Results

### 3.1 Behavioral improvement after TMAS

**Figure 2:**
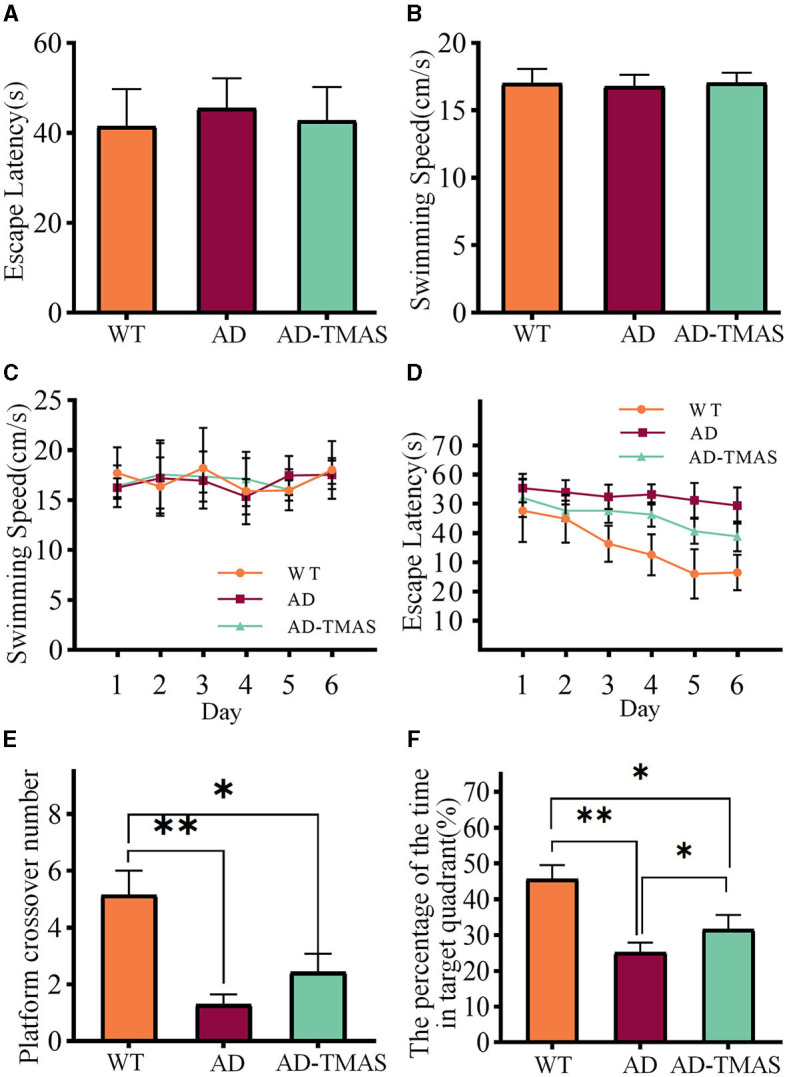
MWM training and test results. **(A)** Escape latencies during visible platform day. **(B)** Swimming speeds during visible platform day. **(C)** Swimming speeds during hidden platform days. **(D)** Escape latencies during hidden platform days. **(E)** Platform crossover number during probe day. **(F)** Percentage of duration spent in the target quadrant. *p < 0.05; **p < 0.01.

The results of spatial memory assessment in various groups of mice using the Morris water maze (MWM) test as shown in Figure 2. During the visible platform training, there were no significant differences in the time taken by mice from different groups to reach the platform [*F*_(2,18)_ = 0.53, *P* > 0.05] and in the swimming speed of mice in different groups [*F*_(2,18)_ = 0.14, *P* > 0.05] (Figures 2A, B). At this stage, the AD mice showed no motor impairment, and their visual acuity was normal. Subsequently, a 6 day hidden platform training was conducted, and the swimming speed (Figure 2C) and time to reach the platform (Figure 2D) of mice from different groups were recorded. The swimming speed of mice in different groups showed no significant changes as the experiment days progressed [*F*_(5,17)_ = 2.40, *P* > 0.05], indicating that the escape latency during the hidden platform phase was negligible influenced by swimming speed. In fact, it was primarily affected by spatial memory capability of the mice. The escape latency of the control group mice was notably lower than that of the AD group mice from the second day. While the escape latency of the control group mice decreased as the experiment days progressed, the AD group mice showed no significant decrease in escape latency. The stimulation group demonstrated a reduced escape latency when compared to the AD group. Finally, a one-day probe trial was conducted to assess the number of crossings through the original platform location (Figure 2E) and the proportion of time spent in the quadrant of the original platform (Figure 2F). During the probe trial, there was a significant difference in the number of crossings between groups [*F*_(2,18)_ = 9.16, *P* < 0.01], with the AD-TMAS group exhibiting a higher mean value compared to the AD group (AD-TMAS: mean ± SEM = 2.43 ± 1.72, AD: mean ± SEM = 1.29 ± 0.95, *p* = 0.48). The proportion of time spent in the target quadrant also showed significant inter-group variation [*F*_(2,18)_ = 14.50, *P* < 0.01], with the AD-TMAS group displaying significant differences compared to the AD group (*P* < 0.05). The WT group mice exhibited higher numbers of platform crossings and a greater proportion of time spent in the target quadrant compared to the other two groups. The spatial learning and memory ability of AD mice was impaired, whereas TMAS demonstrated a certain degree of improvement in the spatial learning and memory deficits of the AD model mice.

### 3.2 Changes in hippocampal SWR after TMAS

**Figure 3:**
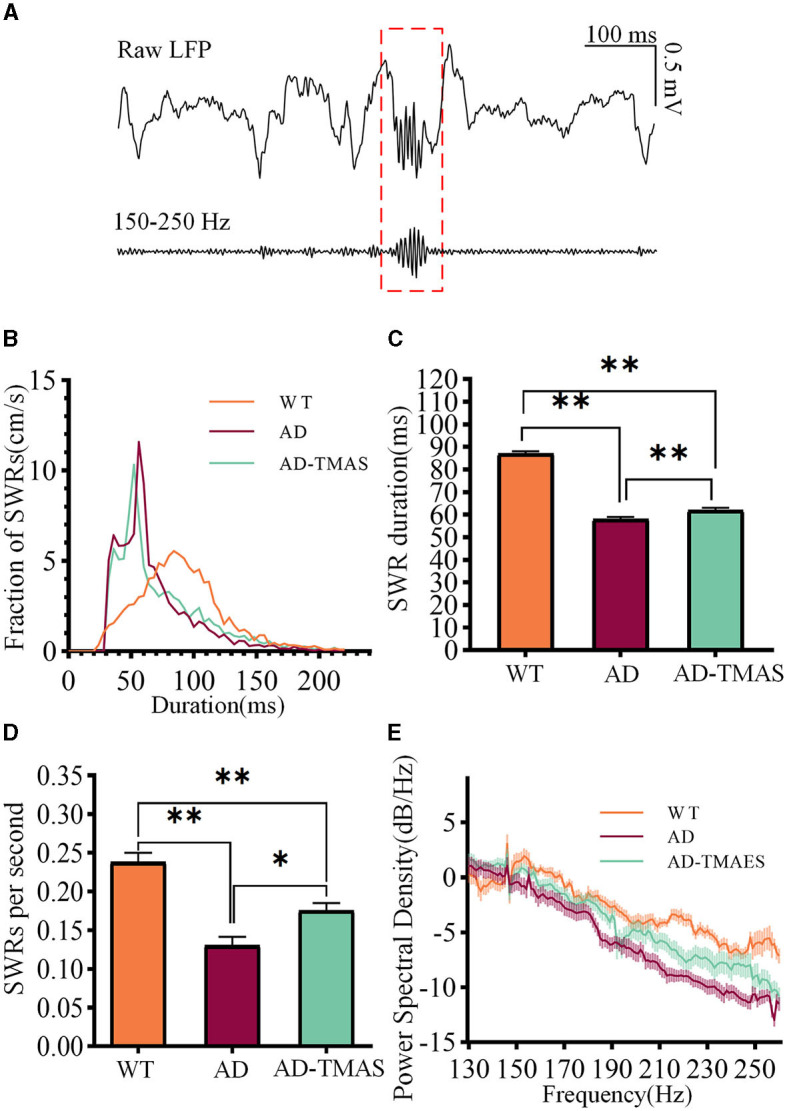
Characteristics of SWRs. **(A)** Representative traces of band-pass filtered SWRs (150 Hz−250 Hz). **(B)** Frequency distribution of SWR durations. **(C)** SWR durations (median with 95% confidence interval). **(D)** Average incidence rate of SWRs. **(E)** Power spectral density of LFPs. The values are presented as mean ± SEM unless otherwise specified; *p < 0.05; **p < 0.01.

SWRs reflect synchronized population activity patterns in the mammalian brain which are crucial for certain aspects of memory functions in the hippocampus. After detecting and recording SWRs (Figure 3A), we examined the characteristics of SWRs. Deficiencies in SWR duration were observed in the AD model mice (Figures 3B, C), with fewer longer duration of SWR compared to the WT group (Kruskal–Wallis test, Bonferroni correction, *P* < 0.01). In comparison to the AD group, TMAS increased the duration of SWRs in the AD-TMAS group (Kruskal–Wallis test, Bonferroni correction, *P* < 0.01). There were significant differences in the incidence rate of SWRs [*F*_(2,18)_ = 23.40, *P* < 0.01] (Figure 3D). *Post-hoc* Scheffe tests indicated that the SWR incidence rate was lower in the AD group compared to the WT and AD-TMAS groups (WT vs. AD, *P* < 0.01; AD vs. AD-TMAS, *P* < 0.05). The AD group exhibited lower power spectral density traces in the LFP frequency range of 150 Hz to 250 Hz compared to the WT and AD-TMAS groups (Figure 3E).

### 3.3 TMAS can modulates slow gamma oscillations during SWRs

**Figure 4:**
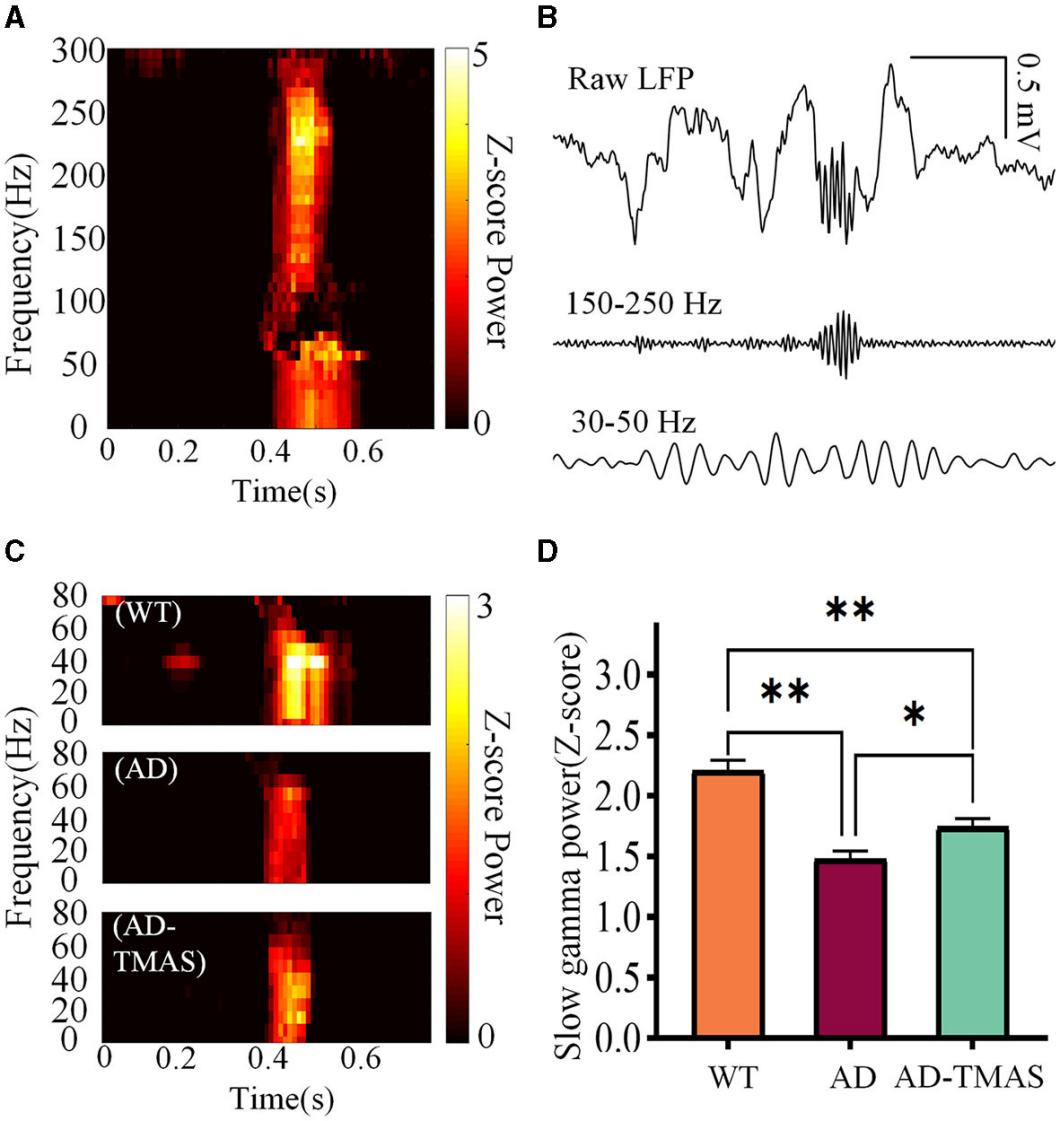
Slow gamma rhythm during SWRs. **(A)** Representative SWR-triggered spectrogram. **(B)** Illustrative trace of slow gamma bandpass filtered signals (30 Hz−50 Hz) during SWRs. **(C)** Representative triggered spectrograms during SWRs for different groups of mice. **(D)** Average SG Z-scores power during SWRs. The values are presented as mean ± SEM; *p < 0.05; **p < 0.01.

The time-frequency diagram revealed an increase in power within the ripple frequency range during SWRs, accompanied by a transient augmentation in slow gamma power (Figure 4A). Then we applied a 30 Hz to 50 Hz band-pass filter to the raw LFP signals (Figure 4B) and computed SWR triggered spectrograms for the 400 ms preceding and following SWRs (Figure 4C). To quantify SG power during SWRs, we calculated the Z-scored power of the SG frequency band (30 Hz−50 Hz) within the first 100 ms after SWR initiation (Figure 4D). Notably, SG power exhibited significant differences during SWRs [*F*_(2,102)_ = 24.56, *P* < 0.01], with AD mice displaying lower SG power compared to WT mice (*P* < 0.01). In comparison to the AD group, the AD-TMAS group demonstrated higher SG power during SWRs (*P* < 0.05).

### 3.4 TMAS enhances phase amplitude coupling in AD mice

**Figure 5:**
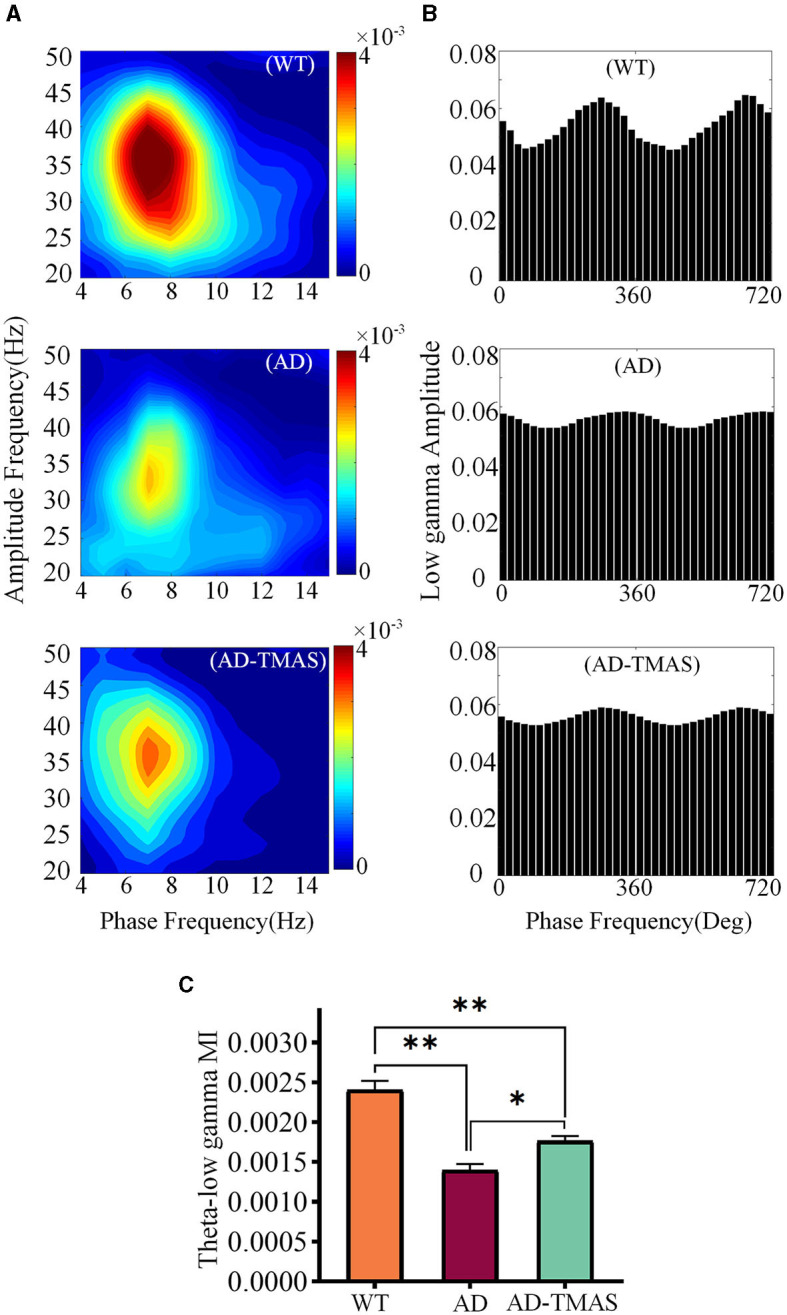
Phase amplitude coupling of hippocampal neural oscillations. **(A)** Example of Phase amplitude coupling of the theta and gamma bands. **(B)** Example of distribution of theta phase and slow gamma amplitude. **(C)** Average modulation index of theta-slow gamma PAC. The values are presented as mean ± SEM; *p < 0.05; **p < 0.01.

In order to assess the synchronization and coordination of neural oscillatory networks, we calculated the phase-amplitude coupling (PAC) between theta oscillations and slow gamma oscillations, where the amplitude of slow gamma oscillations was modulated by the phase of theta oscillations. Subsequently, we computed the modulation index (MI) of PAC. In the WT group, strong theta-gamma cross-frequency coupling was observed (Figures 5A, B). A one-way analysis of variance of the average MI of theta-low gamma PAC revealed significant differences among the groups [Figure 5C; *F*_(2,102)_ = 32.48, *P* < 0.01]. *Post-hoc* tests indicated that the average MI of theta-low gamma PAC in the AD group was statistically lower than in the WT group (*P* < 0.01). Comparatively, TMAS increased the MI of theta-low gamma PAC in the AD-TMAS group when compared to the AD group (*P* < 0.05).

## 4 Discussion

Here, we intervened with a magneto-acoustic couple stimulation that combines static magnetic field and ultrasound in APP/PS1 AD model mice. We found that AD model mice exhibited deficits in the MWM test, and that TMAS improved the results of the MWM test. To investigate the underlying mechanisms, we monitored LFPs in the mouse hippocampus, which reflect neuronal electrophysiological activity. Furthermore, we measured and compared neural oscillations in the hippocampus, an important feature of memory function in the hippocampus. It was found that SWRs features, slow gamma power during SWRs, and theta-gamma PAC were improved by TMAS treatment in the hippocampus of AD model mice.

TMAS is a non-invasive focused neuromodulation technique with the advantages of precision and depth of focused ultrasound stimulation (Zhang et al., 2018; Ning et al., 2022; Xie et al., 2022). TMAS combines the ultrasonic field with the electric field generated by the magneto-acoustic coupling, and the stimulation intensity can be regulated by the ultrasonic parameters. Previous studies have shown that the effect of TMAS is superior to that of transcranial ultrasound stimulation (Zhou et al., 2019; Zhang et al., 2022). We have shown that TMAS can modulate neural oscillatory activity and affect the cross-frequency coupling of LFPs in rats (Dang et al., 2022). The treatment of TMAS on Parkinson's disease model rats also showed beneficial effects of TMAS on learning and memory (Wang et al., 2019). Hence, TMAS has important research value and application prospects for the treatment of neurodegenerative diseases.

The MWM test is a widely employed behavioral paradigm for assessing spatial learning and memory abilities in rodents, demonstrating heightened sensitivity to hippocampal memory impairments (Possin et al., 2016). In our MWM task, the performance of AD group exhibited a pronounced deficit relative to WT group, aligning consistently with prior research outcomes (Luo et al., 2023). During the hidden platform training, AD mice exhibited prolonged latency in locating the platform, indicative of compromised spatial learning abilities in this cohort. Furthermore, impaired memory retention was observed in AD mice, as evidenced by reduced time spent within the target quadrant during the probe test and a diminished preference for the target platform area. In this study, the application of TMAS demonstrated improvements in the performance of AD model mice in the MWM test, suggesting facilitation of spatial learning and memory consolidation.

The hippocampus is a crucial brain region for memory formation and consolidation, coordinating the encoding of episodic memory, spatial navigation, and contextual learning through its normal functioning (Jeffery, 2018). Alzheimer's disease often leads to disruptions in hippocampal function, affecting memory acquisition and recall deficits, thus impacting overall cognitive performance (Dautricourt et al., 2021). In non-invasive neural modulation for AD patients, regions interconnected with the hippocampus are common stimulation targets. The combined approach of transcranial magnetic stimulation and electroencephalography allows for the measurement of cortical hyperexcitability in the Precuneus region of AD patients (Casula et al., 2023), serving as a potentially valuable biomarker. Research on cerebellar-cortical plasticity implies that modulating cerebellar neural activity could be a potential therapeutic strategy for AD patients (Di Lorenzo et al., 2020). Future applications targeting effective stimulation points in humans still require further investigation. Neural oscillations involve rhythmic and coordinated neuronal firing, playing a vital role in memory consolidation and retrieval processes within the hippocampus (Mehak et al., 2022). With its dense population of neurons and distinct interneurons, the hippocampus facilitates long-range synchronization across regions, making it an ideal model system for studying brain rhythms (Ellender and Paulsen, 2010).

Hippocampal SWR, an eruption of hippocampal activity, represents an essential neuronal oscillatory phenomenon that is closely associated with the encoding of memories and the transfer of recently acquired memory traces to the neocortex for long-term storage (Xie et al., 2023). It is suggested that SWRs emerge as a result of synchronized activation of the CA1 region, driven by inputs from hippocampal CA3 pyramidal neurons (Buzsaki, 2015). Place cells within the hippocampus exhibit specific firing patterns when animals occupy distinct locations in their environment (He et al., 2023). These precise firing sequences of place cells can be reactivated (“replayed”) during subsequent SWRs that occur during periods of sleep or wakeful immobility (Zhou and Norimoto, 2023). This implies that SWRs may play a crucial role in recalling and consolidating spatial memories. Hippocampal SWRs facilitate the regulation of synchronized neuronal firing and encourage coordinated activity across brain regions, and impaired coordination may conceivably impair memory processes. Studies have demonstrated that ripple disruption and sleep deprivation after one-session learning in rats affected their performance in maze-based spatial memory tasks, leading to the elimination of long-term memory expression (Aleman-Zapata et al., 2022). Furthermore, research suggests that rats exhibit extended SWR durations under conditions of heightened memory demand, and artificially prolonging SWR duration through optogenetic techniques has been demonstrated to enhance maze learning performance (Fernández-Ruiz et al., 2019).

Neural activity critical for memory can be disrupted thereby leading to neural network dysfunction due to the effects of AD pathology on the hippocampus (Prince et al., 2021; Wu et al., 2023). Anomalies in hippocampal SWRs have been observed in various AD mouse models, potentially offering insights into the mechanisms underlying memory deficits in AD (Sanchez-Aguilera and Quintanilla, 2021; He et al., 2023). *In vivo* studies have indicated a reduced incidence of SWRs in 5xFAD mice (overexpressing amyloid-like proteins) (Iaccarino et al., 2018), rTg4510 mice (overexpressing human Tau protein) (Witton et al., 2016), apoE4-KI mice (knocking in Apolipoprotein E4 gene) (Gillespie et al., 2016), and APP/PS1 mice (overexpressing human genes for amyloid precursor protein and presenilin 1) (Jura et al., 2019). Decreased SWR durations have also been reported in 5xFAD mice (Prince et al., 2021), APP-KI mice (knocking in Amyloid Precursor Protein gene) (Funane et al., 2022), and TgF344 rats (bearing the human APP gene with Swedish mutation and the human PS1 gene with the deltaE9 mutation) (van den Berg et al., 2023). These findings may be attributed to AD pathology inducing disruptions in synaptic plasticity and interneuronal function, thereby interfering with precise spike-timing during SWRs and resulting in shortened SWR durations. In fact, in this study, abnormalities in both the incidence and duration of SWRs were identified, and these aberrations were improved with the application of TMAS.

Gamma oscillations (25 Hz−100 Hz) prominently feature in multiple brain regions, including the hippocampus, exhibiting distinct relevance to cognition and memory processes, and slow gamma (25 Hz−50 Hz) is postulated to enhance memory retrieval by facilitating CA1 input to CA3 (Mably and Colgin, 2018). Slow gamma modulates the timing of neuronal spiking, orchestrates inter-regional communication, and likely serves as a mediator for neural integration and information processing linked to sensory and cognitive functions (Hudson and Jones, 2022). Slow gamma activity in the hippocampus increases during SWRs, and higher coherence and phase locking of slow gamma between CA3 and CA1 correlate with memory replay quality (Jones et al., 2019). Similarly, we found the increase in slow gamma power during SWR in the hippocampus. However, memory-related deficits have been associated with reduced slow gamma during SWRs in several AD mouse models (Stoiljkovic et al., 2019). This decrease in slow gamma during SWRs is thought to be linked to the intricate disruption of synaptic and neuronal function due to the accumulation of Aβ and hyperphosphorylated tau. The deposition of Aβ in the cerebrospinal fluid of AD patients is one of the earliest signs in the progression of Alzheimer's disease. This accompanies neurodegenerative processes and may strongly modulate synaptic efficiency in pathological aging (Martorana et al., 2015). Acetylcholine plays a crucial role in the encoding, consolidation, and retrieval of memory, and disruptions in cholinergic neurotransmission at higher levels may be associated with Aβ levels (Martorana et al., 2014). Cholinergic activity is correlated with human memory tasks and may be modulated through neurophysiological means (Bonni et al., 2017). Our findings indicate a reduced slow gamma activity in the AD group of mice compared to the WT group during SWRs. The AD-TMAS group exhibited an elevation in slow gamma activity during SWRs, potentially linked to the enhancement of neural plasticity through TMAS treatment (Wang et al., 2019).

A reduction in theta-gamma PAC can be observed in both AD patients and AD mouse models, similar to the observations in the current study (van den Berg et al., 2023). An increase in theta-slow gamma PAC was observed in AD model mice after TMAS treatment. The coordination of neuronal activity has been observed not only within neuronal networks and brain regions but also across various frequency bands of neural oscillations (Marzetti et al., 2019). This cross frequency coupling, involving different frequency bands, is considered a fundamental aspect of cognitive function (Yakubov et al., 2022). Such relationships of coupling can unveil interactions among different frequency components of signals, thereby aiding in the comprehension of dynamic properties and information transmission mechanisms of the nervous system. PAC refers to the modulation of the amplitude of high-frequency components of electrophysiological signals by the phase of low-frequency components (Özkurt, 2012). During animal engagement in spatial learning and navigation, research has shown a notable enhancement in theta-gamma PAC power (Kitchigina, 2018). Consequently, the strength of theta-gamma coupling within the hippocampus is typically associated with accurate performance in cognitive tasks.

## 5 Conclusion

In conclusion, our study demonstrates the beneficial effects of TMAS treatment on spatial memory deficits and abnormal neural oscillations in the APP/PS1 transgenic AD mouse model. The memory impairments in AD model mice are possibly associated with defects in hippocampal oscillations, which can be alleviated through TMAS intervention. These findings suggest the potential of rescuing cognitive impairments caused by neurodegenerative diseases through the modulation of brain oscillations via neurostimulation. This study demonstrates that TMAS presents a hopeful opportunity for non-intrusive therapeutic interventions in AD.

## Data availability statement

The original contributions presented in the study are included in the article/supplementary material, further inquiries can be directed to the corresponding author.

## Ethics statement

The animal study was approved by the Biomedical Ethics Committee of the Hebei University of Technology. The study was conducted in accordance with the local legislation and institutional requirements.

## Author contributions

SZ: Data curation, Formal analysis, Investigation, Methodology, Validation, Visualization, Writing—original draft, Writing—review & editing. ZG: Data curation, Formal analysis, Investigation, Methodology, Validation, Visualization, Writing—original draft, Writing—review & editing. YX: Methodology, Writing—review & editing. JM: Methodology, Writing—review & editing, Investigation. JL: Writing—review & editing, Investigation, Methodology. ZL: Investigation, Writing—review & editing, Methodology. XX: Investigation, Methodology, Writing—review & editing. GX: Writing—review & editing, Investigation, Methodology.
